# Flow rate accuracy of infusion devices within healthcare settings: a systematic review

**DOI:** 10.1177/20420986231188602

**Published:** 2023-07-21

**Authors:** Opeyemi Atanda, Jonathan West, Tom Stables, Chris Johnson, Robert Merrifield, James Kinross

**Affiliations:** Department of Surgery and Cancer, Faculty of Medicine, Imperial College London, St. Mary Campus, QEQM Building, London, SW7 2AZ, UK; Flomark Limited, UK; Flomark Limited, UK; Parallaix Limited, UK; Parallaix Limited, UK; Department of Surgery and Cancer, Faculty of Medicine, Imperial College London, St. Mary Campus, QEQM Building, London, SW7 2AZ, UK

**Keywords:** infusion pumps, intravenous drug delivery system, medication safety, patient safety

## Abstract

**Background:**

One in five patients admitted to the hospital treated with intravenous (IV) fluid therapy suffer complications due to inappropriate administration. Errors have been reported in 13–84% of the preparation and administration of IV medications. The safe delivery of IV fluids requires precise rate administration.

**Objectives:**

This systematic review aims to determine the accuracy of infusion sets and devices and examine the factors that affect the flow rate accuracy of devices.

**Data Sources and Methods:**

Six databases (CINAHL, MEDLINE PubMed, EMBASE, Web of Science and Cochrane Database of systematic reviews) were systematically searched. Search terms included infusion pumps, infusion devices, flow rate accuracy, fluid administration rate, gravity-led infusion set and fluid balance. Studies were included if they examined infusion devices’ flow rate accuracy and drop rates for fluids or non-oncological drugs. Findings were tabulated and synthesised qualitatively. The quality of the studies was examined based on the design of the studies due to their heterogeneity.

**Results:**

Eight studies were included: Four studies were conducted on human subjects in the hospital environment; studies recruited 182 participants between the ages of 18 and 94 years. Two studies examined flow rate accuracy in recruited patients across 509 observations and 2387 drip hours. No trials prospectively assessed the accuracy of infusion pumps in the clinical domain, and no studies were reported on patient safety outcomes. Four studies examined the impact of mechanical and physiological factors on the flow rate accuracies of infusion devices. Height and back pressure simulated vibrating conditions, the viscosity of IV fluid and the positions of patients were reported to have a significant impact on infusion volume and flow rates of infusion devices. Additionally, giving sets that vary from the manufacturer’s specifications are reported to increase error percent by 10–20%.

**Conclusion:**

Infusion devices are an important source of error in administering IV fluids. Yet, there needs to be more prospective trial data to support their clinical accuracy and the impact on patient outcomes. Future flow variability and accuracy studies should capture their impact on patient safety and clinical outcomes.

## Plain language summary

Are the flow rate of infusion devices accurate in fluid administration?

Background/Why was this study done?

Nearly all patients in healthcare settings undergo treatment with fluid therapy that is administered through a vein. Inaccurate intravenous fluid administration causes patient harm. However, very little information in the literature explains how precisely intravenous fluid is administered.

What did the researchers do?

We reviewed the literature on flow rate accuracies of specific infusion devices and examined the factors that affect the flow rate accuracies of intravenous fluid administered to patients.

What did the researchers find?

We found that the flow rate accuracies of infusion devices vary greatly, and they are often affected by physiological and mechanical factors. However, the precise impact of this on patients’ clinical outcomes is not often reported, representing a significant knowledge gap.

What do the findings mean?

We conclude that there is an urgent need to improve the reporting and precision of intravenous fluid rate administration and to understand how this impacts patient safety and clinical outcomes.

## Background

NICE guidelines estimate that one in five patients who undergo fluid therapy during admission^1^ to replace insensible fluid losses, maintain fluid intake and correct electrolyte imbalance suffer some form of complication due to inappropriate administration.^2^ Previous studies into the incidence, causes and severity of errors in administering and preparing intravenous (IV) medications have reported errors ranging from 13 to 84%; however, limited studies have reported the severity of these errors.^3,4^ It is well-established that prescribing errors^5–9^ and a lack of staff education are important sources of patient harm in patients receiving IV fluids.

The most basic form of IV infusion utilises the gravity-led set, which involves raising the prescribed IV fluid bag above a patient, allowing sufficient hydrostatic pressure to overcome peripheral pressure in the vein.^10^ The most basic method for assessing flow rate requires counting the number of drops per minute, although more sophisticated automated drip counting measures are commonly used. The accuracy of the infusion delivered *via* the gravity-led sets has been reported as sub-optimal in previous studies.^11,12^ Han *et al.*^13^ their study reported a median deviation of the flow rate of −47 mL/h in infusions administered using roller clamps for infusions prescribed at a rate between 0 and 50 mL/h. Incorrect IV infusion rates have also been reported as the most common error in IV fluid administration. Ensuring the accurate flow of fluids through various infusion devices is crucial for patient safety across all clinical conditions.^13,14^ This is exacerbated by the significant variation in infusion pumps and administration sets utilised across various hospitals.^4^ From a clinical perspective, hypovolemic patients receiving IV fluids at a slower than intended rate may worsen their condition: conversely, fluid overload on patients in specific circumstances like heart failure.^9^

Automated infusion pumps have reduced the failings associated with IV fluid administration.^15^ Infusion pumps provide many features that ensure the accurate administration of drugs to patients; some include alarms, visual infusion rate settings and electronic monitoring.^10,15^ However, infusion pumps have some disadvantages over gravity-led infusion sets.^10^ Alarms are occasionally ignored, and the infusion is interrupted. The variability in the design specifications of the various manufacturers on the accuracy of the IV fluid administered is some of the issues reiterated in previous studies.^10,16^ We hypothesise that infusion pumps significantly contribute to the incidence of medical errors with commonly prescribed fluids and drugs in the ward environment. This review is intended to highlight error rates in the administration rate of IV fluids that cause patient harm and how significant this effect is. Understanding the variability in the flow rate accuracy across various infusions is rarely reported in the literature; this review aims to critically appraise the flow rate accuracy of existing infusion sets and devices reported in the literature and to establish factors that may adversely influence the precision administration of IV fluids.

## Methods

### Protocol and registration

This review was conducted following guidelines outlined in the Preferred Reporting Items for Systematic Reviews and Meta-Analyses (PRISMA),^17^ and the review protocol was registered on the Open Science Framework (https://osf.io/yxwnu/?view_only=091f3ec89e884ed68591f62d08258785). This was not reported in PROSPERO because we were not reporting on any clinical outcome in this review.

### Inclusion and exclusion criteria

The search was limited to original papers written in English only. Reviews, case reports, abstracts and proceedings were excluded. The inclusion criteria for study design were descriptive observational, randomised controlled trials and simulation studies. Inclusion criteria included studies of gravity and infusion devices that have examined IV fluids infusion devices’ flow rate accuracy and the impact of flow rate accuracy on patient-related outcomes.

### Exclusion criteria

Studies that examined smart pumps or syringe infusion pumps alone, especially those that examined the drug library’s compliance rate and users’ behaviour to alert, were excluded. Implementation studies on improving the usability of infusion pumps were also excluded.

### Search strategy

The databases of CINAHL, MEDLINE (Limits – English, adults’ population), PubMed, EMBASE, Web of Science and Cochrane Database of systematic reviews were searched for eligible studies. Searches were conducted from August 2022 to September 2022; the search looked at studies conducted until 2022. A search strategy for each database was developed using the Polyglot Systematic Review accelerator. (Restrictions to only Articles written in English and adults.)

Several initial scoping searches were conducted and discussed with the research team. A few key search terms were extracted and concluded upon.

### Search terms

Search terms included gravity-led infusion set (OR) infusion pumps (OR) infusion devices (AND) flow rate accuracy (OR) fluid administration rate (OR) fluid balance.

### Participants/population

Adult patients engaged in any form of infusion administration therapy across hospital settings. This includes ambulatory services day outpatient centres.

### Intervention(s) and exposure(s)

Full use of infusion pumps or gravity-led infusion set for administering any form of fluid irrespective of their viscosity and ensuring the flow rate accuracy of fluids administered.

### Outcomes

Flow rate accuracy of IV fluid administration and factors that affect flow rate accuracy.

### Data screening

All references were imported into covidence, where duplicates were automatically identified and removed. Initial screening (Abstracts) was conducted by one author (O.A.). Then, all references identified as potentially relevant were obtained in full and transferred to full-text screening. O.A. and J.K. independently screened the full text according to the inclusion and exclusion criteria. Conflicts were resolved in discussions between the reviewers.

### Data extraction and charting process

Following the guidelines suggested by the Centre for Reviews and Dissemination (2009), two reviewers (O.A. and J.K.) extracted data using two purposely developed standardised forms. Disagreement was resolved by consensus. The reviewers developed a data-charting table to extract the outlined data.

The following data were extracted for each included study:

• Title, Author
• Study design/Study origin
• Type of infusion device
• Parameters for the determination of flow rate and accuracy
• Flow rate accuracy reported – This is the average delivery rate selected by the operator (*r*) over the total duration of the test time from start to finish (*T*)
• Manufacturers’ recommendation for the use of the device to ensure accuracy

### Synthesis of results

The included studies were largely heterogenous in design, reporting flow rate accuracy and setting where studies were conducted. To provide a detailed overview, key details of the study were instead extracted from details about the infusion pumps examined in the study, variability of flow rate reported across the study, parameters set across each study to examine flow rate accuracy, the type of fluid used in the study and details about manufacturers recommendation for ensuring flow rate accuracy.

### Risks of bias and quality of individual studies

To address the variability in methods used in the studies we analysed, we utilised a narrative approach with detailed information, evaluated the quality of the studies and minimised potential bias using the Cochrane Collaboration Tool for Bias Risk Assessment. This tool included seven domains: random sequence generation, allocation concealment, blinding of participants and personnel, blinding of outcome assessment, incomplete outcome data, selective reporting and other sources of bias. We assessed each article’s risk of bias and categorised them as high, low, no information or unclear. The Robvis tool (McGuiness, 2019), which is a web application used in the visualisation of risk-of-bias, was used.^18^

## Results

**Figure 1.**
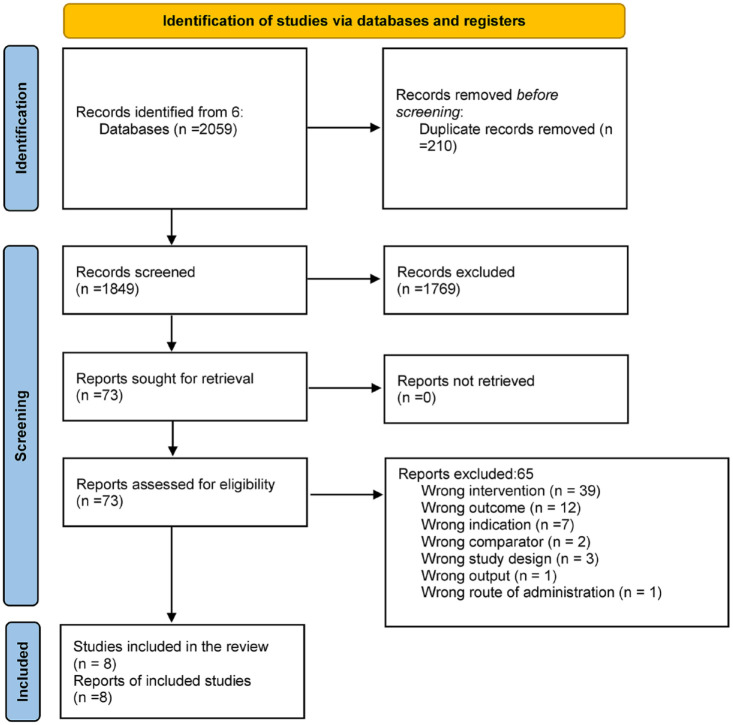
PRISMA flow diagram. Source: Page *et al.*^19^http://www.prisma-statement.org/

The database search resulted in 2059 records. After duplicates were removed, a total of 1849 records remained. A total of 1769 records were excluded in the title and abstract screening, leaving 75 for full-text screening. Of those, one of the papers could not be retrieved, 39 were excluded during the wrong intervention, 12 had the wrong outcome, 5 had the wrong indication, 2 had wrong comparators, 5 had the wrong study design, and 2 had the wrong output and routes of administration. Eight records were qualitatively synthesised. See Figure 1 for the PRISMA diagram. PRISMA checklist is attached as Supplemental material.

### Study characteristics

Only four^20–23^ studies examined flow rate accuracy amongst clinical patients; the number of patients recruited was between 20 and 86 across all the studies. All studies except Simon *et al.*^21^ recruited healthy patients into their study. Fraser *et al.* and Crass *et al.*^20,22^ observed flow rate accuracy in recruited patients across 509 observations and 2387 drip hours, respectively.

Four studies^11,24–26^ examined the flow rate accuracy of infusion pumps using a simulation design. Other studies included one cohort design,^22,27^ one cross-sectional design^23^ and one randomised controlled trial.^20^ The infusion devices examined across the included studies were elastomeric, volumetric, peristaltic, pain/ambulatory, flow regulators, syringes and gravity-led infusion sets. Studies originated from the USA, Australia, Korea, South Africa, Iran, France and Israel.

The parameters for determining flow rate accuracy were outlined across all included studies; infusion duration ranged from 10 min to 27 h across the studies. The fluid used as the infusate for examining the flow rate across the included studies ranges in viscosity; they include distilled water, normal saline, 5% dextrose and 6% hydroxyethyl starch. The viscosity range for IV fluids typically varies between 1 and 15 centipoise (cP), with some specialised fluids having a viscosity as high as 70 cP.^28^ One study^20^ did not state the fluids used. Whilst most of the studies have utilised the recommendations from manufacturers of the various pumps to ensure accuracy, four of the studies^11,22,23^ did not consider that Table 1, attached as Supplementaly material, shows the study characteristics.

### Flow rate accuracy and clinical implications

This review section reports flow rate accuracy across all included studies and the clinical implications of flow rate deviations.

### Flow rate accuracy in clinical domains

Only four^20–23^ studies examined flow rate accuracy amongst clinical patients, Simon *et al.*^21^ examined the peak concentration of amikacin serum in humans, comparing the accuracy of two infusion methods (gravity-fed infusion set *versus* electronic infusion pump). A total of 24 patients with community-acquired pulmonary infections were recruited into the study. Participants were scheduled to receive amikacin by IV route over 1 h with a targeted peak concentration of 35 mg/L. The level of amikacin serum was determined at the end of infusion and 24 h later. The expected concentration *C*_max_ value was significantly lower with gravity than with pump (40.2 ± 12.3 *versus* 50.6 ± 17.6 mg/L, respectively; *p* = 0.04).

Furthermore, Fraser *et al.*^20^ investigated the effectiveness of adding an IV flow device (IVF), which is believed to close the IV line when the container runs empty to a gravity-fed infusion set. The study had four study arms: a roller clamp flow regulator without IVF, a roller clamp flow regulator with IVF, a dial-type regulator without IVF and a dial-type with IVF. Flow rate accuracy was examined as a secondary outcome; there was a significant difference between study arms with an IVF device and the control group (*p* = 0.01). The mean deviation in mL per hour was −5 and −7.2 for the active study arm and −36.0 and −29.7 for the control arm; these are point estimates based on deviations from the prescribed flow rate. Despite examining the reduction of an adverse event as a primary outcome, clinical parameters indicative of an adverse event related to IV fluid administration were not clarified.

The review found only one study^18^ that examined the flow rate accuracy of gravity-flow IV infusion sets using human participants. Drop rates in this study were measured using a drop-rate counter following 509 observations involving 86 patients. Less than 15% of observations were within ±10% of the desired drop rates. Additionally, only 21% of the observations fell within ±20% of the desired drop rates. The study reported changes in patients’ position as a significant influence to drop rates; other factors mentioned included kinking of the tubing and transportation of patients for other hospital procedures. The study did not examine the clinical implication of deviation in desired drop rates.

### Influence of mechanical and physiological factors on flow rates

Four studies^11,23,24,26^ examined the influence of various factors on the flow rate accuracy of the respective pumps examined in their study. Hobbs *et al.*^24^ investigated the impact of height and back pressure on the flow accuracy of infusion pumps. The study reported a significant effect of height, back pressure and pump brand on the mean flow rate (*p*-value < 0.001, 0.003). Hong *et al.*^24^ examined the influence of various vibrating conditions (resting 0 m/s^2^, mild 2 m/s^2^, moderate 6 m/s^2^, extreme 20 m/s^2^) on the flow rate based on a predetermined error range of less than 3% for syringe pumps, 5% for peristaltic pumps and less than 2% for a new generation cylinder pump. Only the new-generation cylinder pump recorded a stable flow rate with less than 2% of the manufacturer-provided error range under all simulated vibrations. A flow rate increase above the error range was reported for the syringe pumps examined for moderate and higher vibrating conditions. Flow rates were reported primarily stable within the known error range for the peristaltic pump; however, overall under-infusion was observed.

Carleton *et al.*^23^ examined the influence of position change of human subjects on flow rate accuracy on five infusion administration devices, including the IVAC 280 controller, an electronic device that was the control, roller clamp device, Dial-A-Flo, 3M IV and Exacdrop. The change in positions examined was supine, sitting and walking positions. The study set a predefined flow rate as 40 ± 4 drops/min. The study reported a significant decrease in drop rates for three of the five infusion administration sets investigated (roller clamp, Dial-A-Flo and Exacdrop) when the position was changed from supine to sitting. At the same time, the position change from sitting to standing did not affect the IVAC 280 and 3M IV but significantly decreased the rates for all other devices. From standing to walking, there was a significant change in the rates of only the roller and Exacdrop devices. The change from walking to the supine position significantly increased the rates for other devices except IVAC 280 and 3M IV.

In the study by Choi *et al.*,^11^ fluids’ viscosity influence on the flow rate accuracy of infusion devices was examined. Four infusion devices (Terufusion, Volumed, Autoclamp and INFUCON) were evaluated; the devices were placed at the same height as the catheter site and were set to deliver 20 ml/h, 40 ml/h, 100 ml/h and 200 ml/h for 3 h. The effect of flow resistance was also examined using a 24-gauge, 19-mm catheter and non-return valve using a crystalloid solution. All infusion devices, except for INFUCON, a gravity-fed infusion set, delivered fluid with high accuracy under all conditions.

### Other studies

Other studies examining the flow rate difference across various infusion pumps were investigated in a laboratory setting. LeRiger^25^ compared the mean value of three elastomeric infusion pumps; the pumps were filled with 200 mL of 0.1% ropivacaine in normal saline. Each was set to infuse at 12 mL/h; they were allowed to run for 10 h with fluid output measured every hour. The study reported the On-Q pump to have infused faster than the set rate [17.3 mL/h; standard deviation (SD) 0.62]. The Baxter pump’s mean output was 10.1 mL/h with a SD of 0.17, narrowly outside the manufacturer’s stated range of 8.0–9.8 mL/h. The Ambu pump’s mean output was 9.5 mL/h with a SD of 0.0089.

### Infusion pump characteristics

In this section, Supplemental Table 2 shows the characteristics of the various infusion pumps examined in the included studies. The characteristics would include specific manufacturer recommendations for users to ensure the flow rate accuracy of the various infusion devices.

The most common infusion devices examined across the included studies were the elastomeric and volumetric pumps. Elastomeric pumps are reported to offer a safer alternative to electronic pumps because they are intended for single-use purposes. The pumping function operates by the continuous contraction of an elastomeric balloon filled with the intended fluid; it is also attached to a flow restrictor that limits the flow rate of the fluid by the difference in pressure between its inlet and outlet.^29^ Flow rate is achieved by one or more of these restrictors attached to the infusion line.

Volumetric pumps are the most common type of infusion pump used in the healthcare setting and were commonly examined in the included studies. They use typical linear or rotary peristaltic pumping under pressure and resistance.^30^ The safety standards of these pumps are reported to follow international guidelines like the IEC60601-2-24; infusion pumps are required to ensure flow rate accuracy of delivery considering all factors like the type of fluid administered, the dosage of the fluid, different infusion rates and different environmental conditions.

Furthermore, the peristaltic pump works by either two shoes or rollers rotating on a wheel inside the pump, forcing the quantity of fluid required. The pumps are reported not to need any priming as they are capable of self-priming; fluid viscosity does not influence fluid transport. But there are downfalls to peristaltic pumps. They are expected to be calibrated to reach acceptable accuracy due to deviation caused through production and the replaceable tubing. Also, the flow rate is sensitive to various differential pressure conditions.^31^

Hong *et al.*^32^ examined a new-generation cylinder pump in their simulation study; the pump combines the advantages of both infusion and syringe pumps based on the principle of sucking and discharging fluid *via* two rotating pump pistons in a cylinder cartridge. In addition, syringe pumps were also investigated in some of the included studies. Although studies examining syringe pumps as a standalone were excluded from this review, it is worth highlighting them because some studies have included them as a comparison against other infusion pumps. One problem with using syringe pumps is the potential of administering unintended bolus injections affected by external environmental factors.^33^

## Quality and risk of bias

**Figure 2.**
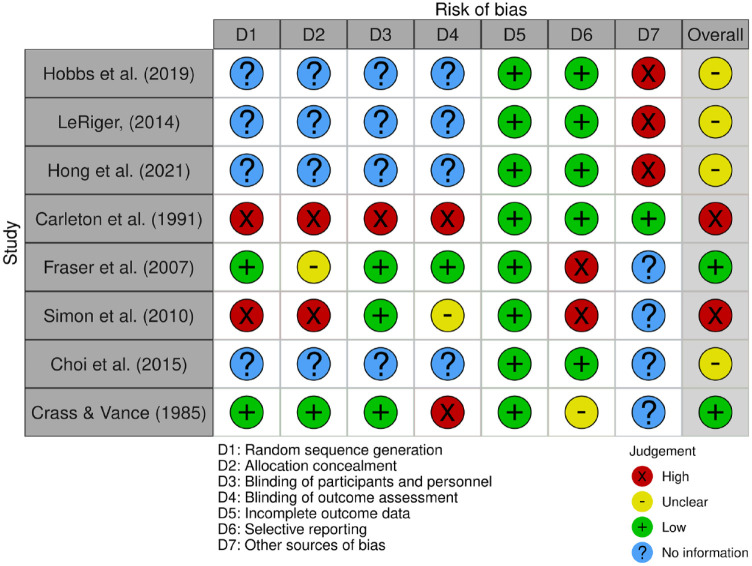
Quality of studies and risk of bias.

Studies were evaluated for their risk of bias and quality. The quality of the evidence was examined according to the design of the studies due to the heterogeneity in the designs of the included studies. The Robvis ‘Generic’ tool showed that two studies had a high risk of bias^23,27^ (see Figure 2 below). Carleton *et al.*^23^ did well in reporting outcomes and potential confounding variables that could affect the results of their study, the article had problems in the ‘random sequence generation’, ‘allocation concealment’, ‘blinding of participants or personnel’ and ‘blinding of outcome assessment’. Simon *et al.*^27^ had problems in the ‘random sequence generation’ and ‘allocation concealment’ domains. Studies that have utilised a simulation design are difficult to rely upon because of their difficulty predicting responses in real-life contexts. For example, one of the studies^24,25^ pointed out the difficulties in creating a similar ambient temperature in a study environment based on standard testing instructions. However, this might not apply to home/hospital environments where infusion pumps are commonly used.

## Discussion

This systematic review aimed to examine the flow rate accuracy of existing infusion devices and factors contributing to the flow rate variability. Eight studies were included, and the studies ranged in designs and settings where they were conducted.

Four studies examined the influence of various external factors on flow rate accuracy. There was a significant effect of height and back pressure on the mean flow rate of the infusion pumps examined. In various vibrating conditions commonly experienced in ambulatory settings, the flow rate was reported to have increased above the error range for syringe pumps. Infusion devices were examined when positions were changed, and roller clamp and Exacdrop devices reported significant changes in flow rates. Also, a study reported a high level of accuracy of all infusion devices examined to administer fluid except the gravity-led device.

Similarly, the concentration of amikacin serum in the blood after an infusion process was significantly lower in gravity-led pumps than in a specific electronic infusion pump (IVAC 589), despite the overarching report of flow rate accuracy with the error range for infusion pumps across the included studies.

Flow rate accuracy is reported to be commonly affected by various factors; some of these factors reported across the included studies include the use of wrong tubing sets different from manufacturers’ specifications, the position of the drip detector to the injection site is reported to increase error percentage by 25% and the use of low-quality batteries.

In many of the studies, an electronic drop counter was used. This has the above inaccuracies but is primarily prone to the fact that drop size itself varies. Gravity administration sets must adhere to ISO8536-4, which states that 20 drops of distilled water must equal 1 mL at a rate of 50 drops per minute; this standard also gives a tolerance of ± 0.1 mL. Thus, gravity administration sets may only be accurate to within 10%. Drop size also varies with changes in flow rate, temperature and fluid viscosity. Given that electronic drop detection is often the means of determining accuracy in certain studies, there is an extra error introduced which complicates findings.

Usability is also a key factor. The practice of determining flow rate by counting drops is rarely used in high resource settings with electronic infusion devices, where flow rates can be set and trusted to be maintained automatically. Circumstances may arise when such equipment is not available, and gravity infusion is used. If the operator is unfamiliar with the practice of counting drops, it is unlikely that the rate will be set correctly.

Conversely, the user interface of electronic infusion devices vary greatly and require specific training. Even taking this into account, there are studies to show infusion errors specifically caused by usability error with the device interface.^34^ The National Patient Safety Agency (NPSA) produced design guidance to help address these issues.^35^

Previous studies have highlighted factors influencing flow rates in infusion devices. In elastomeric pumps, the factors include the cross-sectional area of the flow restrictor, length of the flow restrictor, temperature (at the restrictor and the pump itself), the viscosity of the medication and pressure between the balloon reservoir and the patient connection. Also, volumetric pumps’ flow rate accuracies depend on several factors, for example, environmental conditions. The peristaltic pumps are prone to deviation in flow rate due to the constant need to recalibrate.

With IV infusion pumps, there are still reported concerns regarding the infusion rates and infusion times across the various pumps. These concerns were reiterated in the review conducted by Ko *et al.*^28^ This review has highlighted key factors that influence the flow rate accuracies of the infusion pumps investigated. Therefore, any development of infusion devices needs to consider some of these factors to limit the variability in the flow rate of IV fluids.

IV infusions pose risks to patient safety due to the number of steps involved in administering fluids. The NHS recognises that IV medications pose a challenge for patients and have developed several guidelines to reduce adverse events.^1^ Previous studies have reported that IV infusion is associated with approximately 60% of adverse drug events in healthcare settings.^29^ Accurate fluid management is essential in most areas of healthcare; for example, in perioperative patient care, excessive intraoperative fluid volume due to variability in flow rate can result in oedema or organ dysfunction, whereas hypoperfusion and organ ischemia could result from inadequate fluid volume. Therefore, when examining infusion devices, it is essential to capture any variability in their flow rate and its impact on patient safety and clinical outcomes.

Although this review has highlighted the need for developing infusion devices that can offer accurate infusion rates regardless of earlier stated factors, the review has some limitations. Our focus was mainly on studies that reported flow rate accuracies of infusion devices in adult healthcare settings; this is a very niche area of investigation compared to other areas like paediatrics, which might have reduced our opportunity for synthesis. Also, design heterogeneity is expected in this field, making it challenging to capture the incidence of flow rate accuracy together.

**Figure 3.**
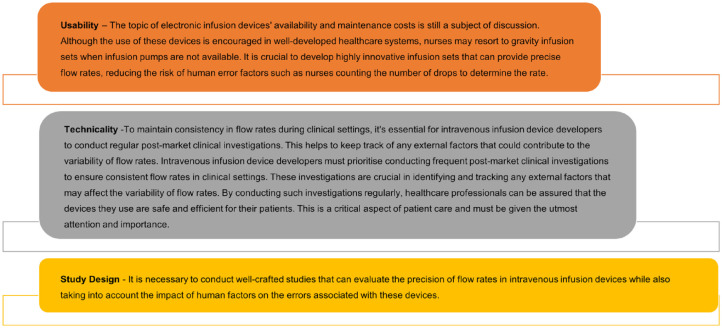
Recommendations following review.

To conclude, there is a need for well-designed prospective trials to investigate the flow rate accuracy of infusion devices; it is also essential to consider the impact of the variabilities of the flow rates on patient safety by linking them to clinical outcomes. Recommendations are made from three different perspective and included in Figure 3.

## Supplemental Material

sj-docx-1-taw-10.1177_20420986231188602 – Supplemental material for Flow rate accuracy of infusion devices within healthcare settings: a systematic review Click here for additional data file. Supplemental material, sj-docx-1-taw-10.1177_20420986231188602 for Flow rate accuracy of infusion devices within healthcare settings: a systematic review by Opeyemi Atanda, Jonathan West, Tom Stables, Chris Johnson, Robert Merrifield and James Kinross in Therapeutic Advances in Drug Safety

## Declarations

**Ethics approval and consent to participate:** Our study did not require ethical board approval because it is a systematic review of published articles and did not involve human participation.

**Consent for publication:** Not applicable.

**Availability of data and materials:** We have, prior to this submission, submitted a review protocol for purpose of integrity, documenting the process we have undertaken for this review unto the Open Science Framework (OSF). (View-only link: https://osf.io/yxwnu/?view_only=091f3ec89e884ed68591f62d08258785)
