# Supplementary material for: Flow rate accuracy of infusion devices within healthcare settings: a systematic review
Source: Ther Adv Drug Saf. 2023 Jul 21;14:20420986231188602. doi: 10.1177/20420986231188602 (PMC10363896; doi:10.1177/20420986231188602)
Supplement: sj-docx-1-taw-10.1177_20420986231188602 – Supplemental material for Flow rate accuracy of infusion devices within healthcare settings: a systematic review [file sj-docx-1-taw-10.1177_20420986231188602.docx]

| **Author** | **Study design** | **Infusion devices** | **Parameters for the determination of flow rate** | **Mean flow rate accuracy reported over a set time period** | | | | | | | | | | |
| --- | --- | --- | --- | --- | --- | --- | --- | --- | --- | --- | --- | --- | --- | --- |
| Hobbs et al. (2019) | Simulation study | - Elastomeric pumps (EP) - Electronic peristaltic pump (EPP) | - All pumps had an expected delivery duration of between 24 and 27 hours. - Total volume (mL) delivered at the infusion endpoint stated by the manufacturer (27hrs B. Braun and 24hrs for the other pumps) - Average flow rate until the infusion endpoint stated by the manufacturer (mL/hr). - The infusion duration, where the infusion endpoint was the point at which the flow rate declined to <10% of the set flow rate specified by the manufacturer and the average flow rate over time. - Peak flow rate of the infusion pump   Percentage of infusion time spent greater than, within and below the accuracy range stated by the manufacturer | **Operating conditions df F p-value** | | | | | | | | | | |
|  |  |  |  | Infusion pump brand 4 29.74 < 0.001 | | | | | | | | | | |
|  |  |  |  | Height 4 8.47 < 0.001 | | | | | | | | | | |
|  |  |  |  | Back Pressure 3 17.26 <0.001 | | | | | | | | | | |
|  |  |  |  | Infusion pump brand x Height 16 3.40 <0.001 | | | | | | | | | | |
|  |  |  |  | Infusion pump brand x back pressure 12 2.62 0.003 | | | | | | | | | | |
|  |  |  |  | Height x back pressure 2 0.16 0.855 | | | | | | | | | | |
|  |  |  |  | Infusion pump brand x back pressure x Height 8 0.74 0.66 | | | | | | | | | | |
| LeRiger, (2014) | Simulation study | - Pain Pump - Multirate portable elastomeric infusion system   Elastomeric infusion device | The examined infusion pumps deliver medication at a flow rate determined by the pressure in the elastomeric reservoir, the flow restriction in the infusion circuit, and the fluid's viscosity.  The constant pressure determines the flow rate of these devices in the elastomeric membrane of the reservoir coupled with various flow control devices.  To adjust for the differences in operating temperature, the nominal flow rate was adjusted for the Baxter and On-Q pumps. The adjusted nominal flow rate was 8.9 mL/h for both. Because the Ambu pump is designed to operate at room temperature, the nominal flow rate did not require adjustment. | **Pump** | | | | | | **No** | **Mean** | | | **P-Value** |
|  |  |  |  | On-Q | | | | | | 10 | 17.3 | | | <0.0001 |
|  |  |  |  | Baxter | | | | | | 10 | 10.1 | | | <0.0001 |
|  |  |  |  | AMBU | | | | | | 10 | 9.5 | | | <0.0001 |
|  |  |  |  |  | | | | | | | | | | |
| Hong et al. (2021) | Simulation study | - Syringe Pumps (SP) - Conventional peristaltic pump (CPP) - New-generation cylinder pump (CYP) | - The infusion system, composed of a 50 ml syringe (Shinchang Medical Co, Korea), was filled with normal saline installed in the infusion pump; it was connected to the infusion analyser (IDA 4 Plus) through an intravenous tubing line and three-way stopcock. The infusion pump was positioned on the vibrating table. - The average flow rate delivered by each infusion pump with infusion settings of 1 ml/h and 5 ml/h was measured over 1 h. - Each experiment was performed for 1 h to analyse the flow delivered to the infusion pump analyser with fluid infusion rate settings of 1 ml/h or 5 ml/h. - Experiment was repeated under different simulated vibrations, including resting state (0 m/s^2^), mild vibration (2 m/s^2^), moderate vibration (6 m/s^2^) and extreme vibration (20 m/s^2^) – Vibration levels were measured using a vibration meter (VM-6360). | Infusion Rate | | | | Syringe Pump (Kabi) | | Syringe Pump (Terumo) | Peristaltic Pump (Arcomed) | | | Cylinder Pump (Meintech) |
|  |  |  |  | Rate at 1 ml/hr | | | | | | | | | | |
|  |  |  |  | Resting (0m/s^2^) | | | | 1.01 | | 1.03 | 0.96 | | | 0.98 |
|  |  |  |  | Mild (2 m/s^2^) | | | | 1.01 | | 1.01 | 0.96 | | | 0.99 |
|  |  |  |  | Moderate (6 m/s^2^) | | | | 1.01 | | 1.03 | 0.93 | | | 0.99 |
|  |  |  |  | Inadvertent bolus at extreme vibrations (20 m/s^2^) | | | | Yes | | Yes | No | | | No |
|  |  |  |  | **Rate at 5 ml/hr** | | | | | | | | | | |
|  |  |  |  | Resting (0m/s^2^) | | | | 5.10 | | 5.04 | 4.78 | | | 4.96 |
|  |  |  |  | Mild (2 m/s^2^) | | | | 5.03 | | 5.05 | 4.79 | | | 4.92 |
|  |  |  |  | Moderate (6 m/s^2^) | | | | 5.25 | | 5.40 | 4.77 | | | 4.95 |
|  |  |  |  | Inadvertent bolus at extreme vibrations (20 m/s^2^) | | | | Yes | | Yes | No | | | No |
| Carleton et al. (1991) | Cross-sectional Study | - Volumetric Pump - IV extension set - Exadrop - Flow regulator | - Drip rates were measured by two study members who counted and recorded. - Visual counting of drops: One research team member counted the drops, and the other assembled the IV set and calibrated it to 40 drops/min. - Time zero was considered the start point where the IV set had been assembled and the drip rate set calibrated to 40 drops/min. - Participants remained in a specified position for 10, and the drip rate was counted at minutes 0, 4 and 9 for one minute each. - While the participant moved into a new position, the drop count continued for two minutes (two one-minute intervals). - Participants spend three minutes in each position at the end of each sequence, where the drop count continued (three one-minute intervals). - The drip rate was reset to 40 drops/min after each position change was complete. - If the drip rate fell below 10 drops/min after a position change, the pole height was adjusted to re-establish a flow rate of 40 drops/min. - Accuracy was defined as maintaining a flow rate of 40 ± 4 drops/min. |  | | | IVAC  280 | Roller Clamp | | 3M IV Flow regulator | Dial-A-Flo | | | Exacdrop |
|  |  |  |  | Before position change | | | 41.17 | 38.17 | | 39.67 | 40.17 | | | 40.08 |
|  |  |  |  | After Position change | | | | | | | | | | |
|  |  |  |  | Supine to sitting | | | 41 | 27 | | 40 | 32 | | | 30 |
|  |  |  |  | Sitting to standing | | | 41 | 23 | | 40 | 25 | | | 30 |
|  |  |  |  | Standing to walk | | | 42 | 37 | | 39 | 39 | | | 36 |
|  |  |  |  | Walking to supine | | | 41 | 76 | | 40 | 72 | | | 72 |
| Fraser et al. (2007) | Randomised Controlled Trial | - Flow regulator | - The primary variable was efficiency, as indicated by the reduction in adverse effects.   The secondary variables were flow rate accuracy and cost implications (AE-related wastage). |  | | **n** | | **Deviation (ml per hour)** | | **SD** | | **SE** | | |
|  |  |  |  | Standard/roller clamp | | 68 | | -29.7 | | 50.9 | | 6.2 | | |
|  |  |  |  | IVF/Roller clamp | | 78 | | -5 | | 60.6 | | 6.9 | | |
|  |  |  |  | Standard/dial-a-flow | | 38 | | -36 | | 35.5 | | 5.8 | | |
|  |  |  |  | IVF/Dial-a-flow | | 14 | | | -7.2 | 75.9 | | 20.3 | | |
| Simon et al. (2010) | Cohort study | - Gravity-fed infusion set. - Volumetric pump | - Blood samples (5 mL) were collected into an additive-free aluminium-foil-protected tube from the opposite arm at the end of infusion (T_inf_) and 24 h later (T_24_). - No consensus exists for once-daily aminoglycoside therapy target concentrations. In various published data, the reported mean amikacin peak concentrations (30e60 min after infusion) are included within the range of 28-45 mg/L.   The target concentration was placed at 35 mg/L in this study. |  | | Gravity-led | | | Pump-controlled | P-value | | | | |
|  |  |  |  | C_max_ (mg/L) | | 40.2 | | | 50.6 | 0.04 | | | | |
|  |  |  |  | Volume delivered | | 0.33 | | | 0.27 | 0.07 | | | | |
|  |  |  |  | T_1/2_ (h) | | 5.23 | | | 4.83 | 0.49 | | | | |
|  |  |  |  | CL (L/h/kg) | | 0.05 | | | 0.05 | 0.54 | | | | |
| Choi et al. (2015) | Simulation study | - Peristaltic (PP) and volumetric pumps (VP). | - Gravimetric   method of measuring fluid volumes was selected.  rather than depending on the visual reading of a fluid meniscus to ensure flow accuracy is captured.   - Infusion devices were placed 80 cm above the catheter insertion sites and were set to deliver 20 ml/h, 40 ml/h, 100 ml/h, and 200 ml/h for three h: 100 ml, 300 ml, 500 ml, and 800 ml beakers were used according to infusion velocity, respectively. | **Infusate** | **Flow rate (ml/hr)** | **Terfusion** | | | **Volumed** | **AutoClamp** | | | **Infucon** | |
|  |  |  |  | Crystalloid / c 18G catheter | 20  40  100  200 | 98.33  98.19  96.72  97.11 | | | 98.89  99.58  98.06  98.97 | 100.00  99.86  99.33  99.33 | | | 110.28  107.08  108.33  110.22 | |
|  |  |  |  | Crystalloid/c 24 G catheter | 20  40  100  200 | 98.06  98.06  96.67  97.08 | | | 98.61  99.44  95.83  98.94 | 99.72  99.72  99.28  99.31 | | | 109.17  106.53  108.11  109.96 | |
|  |  |  |  | Crystalloid/ non-return valve | 20  40  100  200 | 97.97  97.34  97.67  97.58 | | | 97.21  97.44  96.89  97.34 | 98.34  98.79  98.28  98.38 | | | 109.17  106.53  108.11  109.96 | |
|  |  |  |  | Colloid | 20  40  100  200 | 100.83  101.39  99.94  100.50 | | | 100.83  101.25  100.11  100.17 | 102.50  103.19  101.17  101.42 | | | 62.22  60.14  61.39  58.14 | |
|  |  |  |  | D20W | 20  40  100  200 | 98.84  98.38  98.44  98.24 | | | 98.93  98.15  98.23  98.23 | 101.20  101.20  101.12  101.12 | | | 82.22  80.19  81.39  82.15 | |
| Crass & Vance (1985) | Cohort Study | Gravity-flow IV infusion systems | Drop rates were measured using a drop rate counter (IMED 340). The device can record 0-99 drops/minute. The study collected data over 15hrs daily, approximately every two hours between 04:00 and 19:00.  Prescribed IV flow rate, type of infusion, type of IV set used, drop rate and volume of fluid remaining were recorded. | Flow Rate (ml/hr) | No. observations | Volume Delivered(ml) | | | | Mean % difference | | | | |
|  |  |  |  |  |  | Desired | | | Measured |  |  |  |  |  |
|  |  |  |  | 30  50  75  100  125  150 | 22  33  23  85  36  22 | 57.6  95.7  148.4  192.8  240.5  293.2 | | | 76.8  116.2  122.6  153.4  118.1  194.8 | 33.3  21.4  -17.4  -20.4  -21.8  -33.6 | | | | |

**Table 1: Study characteristics and results description**

|  | **Title** | **Infusion devices** | **Manufacturers’ recommendation for the use of the device to ensure accuracy** |
| --- | --- | --- | --- |
|  | Hobbs et al. (2019) | - Elastomeric pumps (EP) - Electronic peristaltic pump (EPP) | - Elastomeric pump manufacturers recommend that the restrictor element is taped to the patient’s skin. This is to maintain a restrictor temperature of 31–32°C during an infusion. - Manufacturers typically advise that elastomeric infusion pumps should be kept at the same height as the infusion site (e.g., 0cm difference in height) to facilitate accurate infusion flow rates per the international standard. - Peristaltic electronic pump is not expected to be affected by any changes in infusion bag properties. |
|  | LeRiger (2014) | - Pain Pump - Multirate portable elastomeric infusion system - Elastomeric infusion device | - The manufacturer-provided references specify the accuracy to be ± 15%-20% from nominal for the On-Q pump, ± 10% for the Baxter pump, and ± 15% for the Ambu pump. - The pumps vary in the manufacturer’s recommendations for temperature management. Baxter pumps are designed to operate at a temperature of 31.1⁰c. The flow rate will decrease by 2.3% per one degree Celsius and should be accurate within ± 10%. - The On-Q pump does specify that it is designed to operate while in contact with the patient’s skin at a temperature of 31°C.The package insert states flow rate will decrease by 1.4% per 0.6°C in temperature. This should be accurate within plus or minus 15% to 20%. - Ambu pump is designed to operate between 20°C and 24°C it must be assumed that the flow regulator need not be secured directly to the patient’s skin. The manufacturer states accuracy is within 5% for the Ambu pump. |
|  | Hong et al. (2021) | Syringe Pumps (SP)  Conventional peristaltic pump (CPP)  New-generation cylinder pump (CYP) | - Changes in flow rate were considered significant if the error ranges increased beyond the known (manufacturer-provided) error ranges. The author didn’t give specific details of the error range for each pump. - Study used a generic error range commonly reported in the literature as less than 3% for syringe pumps and 5% for peristaltic pumps. |
|  | Carleton et al. (1991) | - Volumetric Pump - IV extension set - Exadrop - Flow regulator | No mention of the manufacturer’s guideline utilised in the study to determine rate accuracy. |
|  | Fraser et al. (2007) | Flow regulator | - The IVF device closes the IV line when a container runs empty. The line remains free of air, and the fluid column is maintained. Reverse blood flow should be limited, the formation of blood clots reduced, and air emboli prevented. The inclusion of the IVF maintains the set flow rate by creating a new hydraulic head reference point and keeping it constant for the infusion duration. |
|  | Simon et al. (2010) | - Gravity-fed infusion set. - Volumetric pump - Specific infusion device? Name not given. | No mention of the manufacturer’s guideline utilised in the study to determine rate accuracy. |
|  | Choi et al. (2015) | Peristaltic (PP) and volumetric pumps (VP). | - The peristaltic pumps have a row of horizontally placed fingers that sequentially compress the intravenous tubing in a wavelike manner. Accuracy is reported to be within 5-10% of the selected flow rate. - The volumetric pump (Terufusion and Volumed) examined in the study also used peristaltic system. - AutoClamp is a positive-pressure device that uses a peristaltic system. |
|  | Crass & Vance (1985) | Gravity-flow IV infusion systems | No mention of the manufacturer’s guideline utilised in the study to determine rate accuracy. |

**Table 2: Pump description and manufacturers determination of flow rate accuracy.**
